# Supplementary material for: Competitive interactions affect introgression and population viability amidst maladaptive hybridization
Source: Evol Appl. 2024 Jul 1;17(7):e13746. doi: 10.1111/eva.13746 (PMC11217556; doi:10.1111/eva.13746)
Supplement: Supplementary file 1 — Figures S1–S7. [file EVA-17-e13746-s001.zip › eva13746-sup-0001-Figure_S1.pdf]

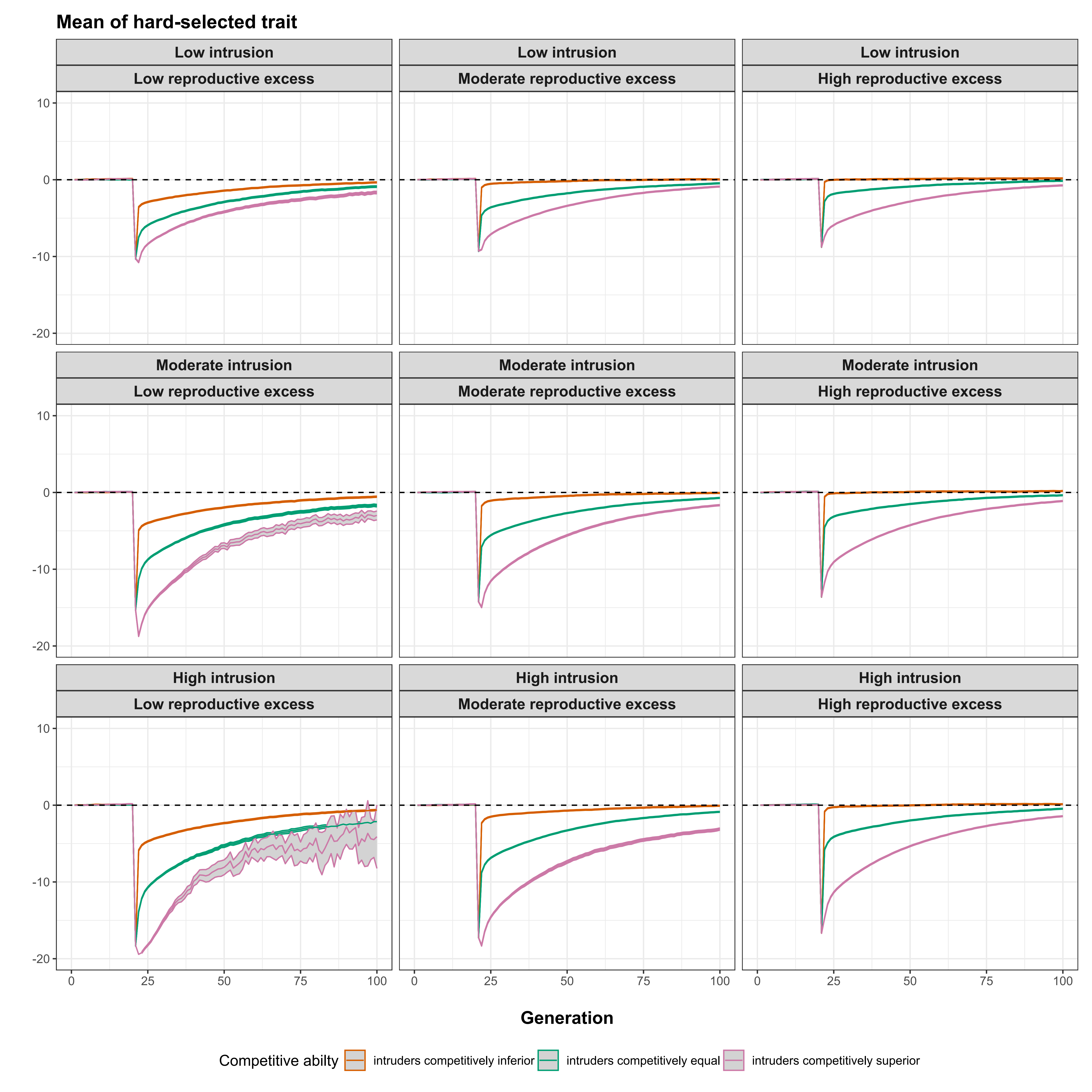

Mean of hard-selected trait
Low intrusion
Low reproductive excess
Low intrusion
Moderate reproductive excess
Low intrusion
High reproductive excess
Moderate intrusion
Low reproductive excess
Moderate intrusion
Moderate reproductive excess
Moderate intrusion
High reproductive excess
High intrusion
Low reproductive excess
High intrusion
Moderate reproductive excess
High intrusion
High reproductive excess
10
0
-10
-20
0
25
50
75
100
Generation
Competitive abilty
intruders competitively inferior
intruders competitively equal
intruders competitively superior
