# Supplementary material for: Competitive interactions affect introgression and population viability amidst maladaptive hybridization
Source: Evol Appl. 2024 Jul 1;17(7):e13746. doi: 10.1111/eva.13746 (PMC11217556; doi:10.1111/eva.13746)
Supplement: Supplementary file 1 — Figures S1–S7. [file EVA-17-e13746-s001.zip › eva13746-sup-0004-Figure_S4.pdf]

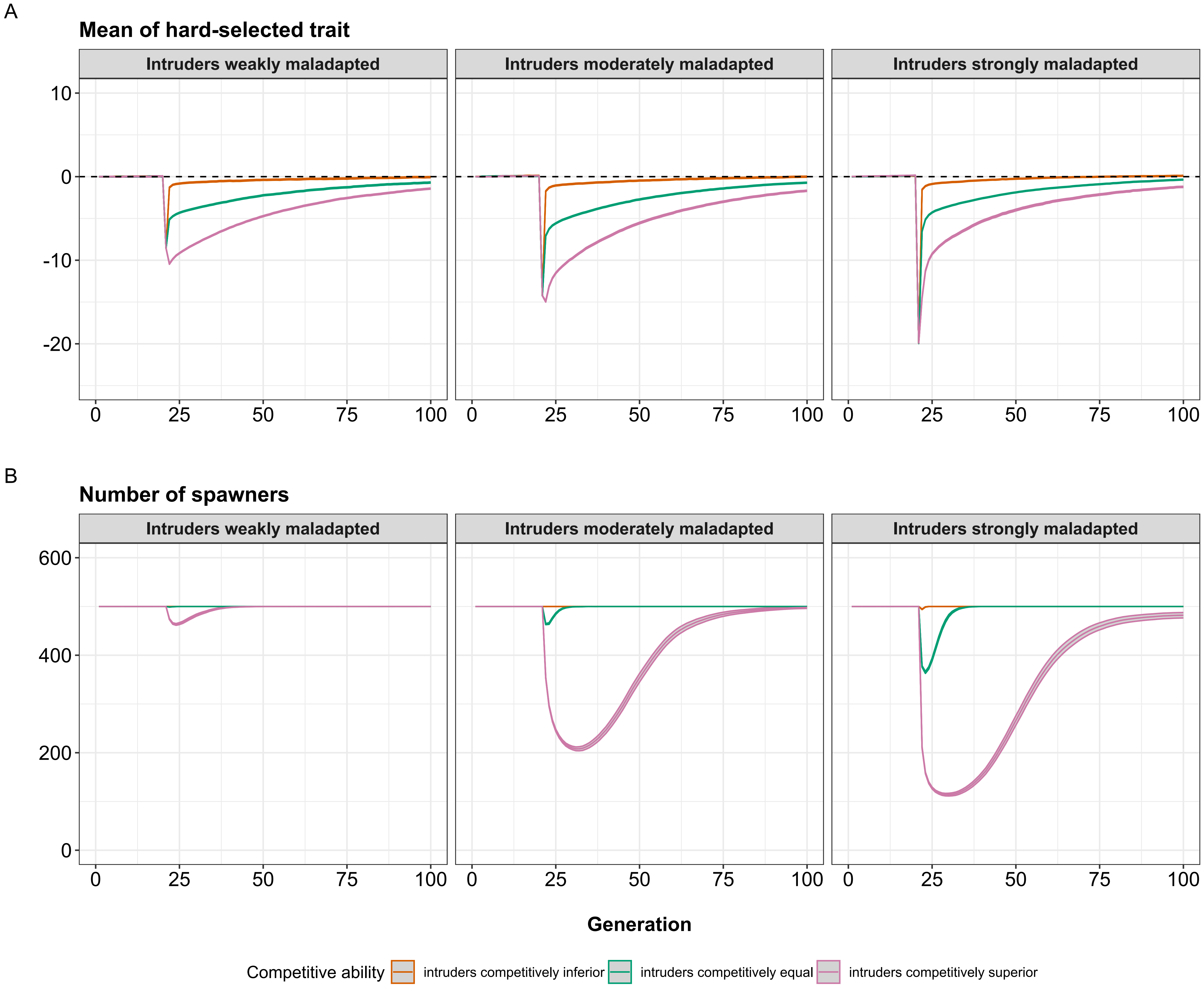
A
Mean of hard-selected trait
Intruders weakly maladapted
Intruders moderately maladapted
Intruders strongly maladapted
10
0
-10
-20
0
25
50
75
100
B
Number of spawners
Intruders weakly maladapted
Intruders moderately maladapted
Intruders strongly maladapted
600
400
200
0
Generation
Competitive ability
intruders competitively inferior
intruders competitively equal
intruders competitively superior
