# Supplementary material for: Competitive interactions affect introgression and population viability amidst maladaptive hybridization
Source: Evol Appl. 2024 Jul 1;17(7):e13746. doi: 10.1111/eva.13746 (PMC11217556; doi:10.1111/eva.13746)
Supplement: Supplementary file 1 — Figures S1–S7. [file EVA-17-e13746-s001.zip › eva13746-sup-0006-Figure_S6.pdf]

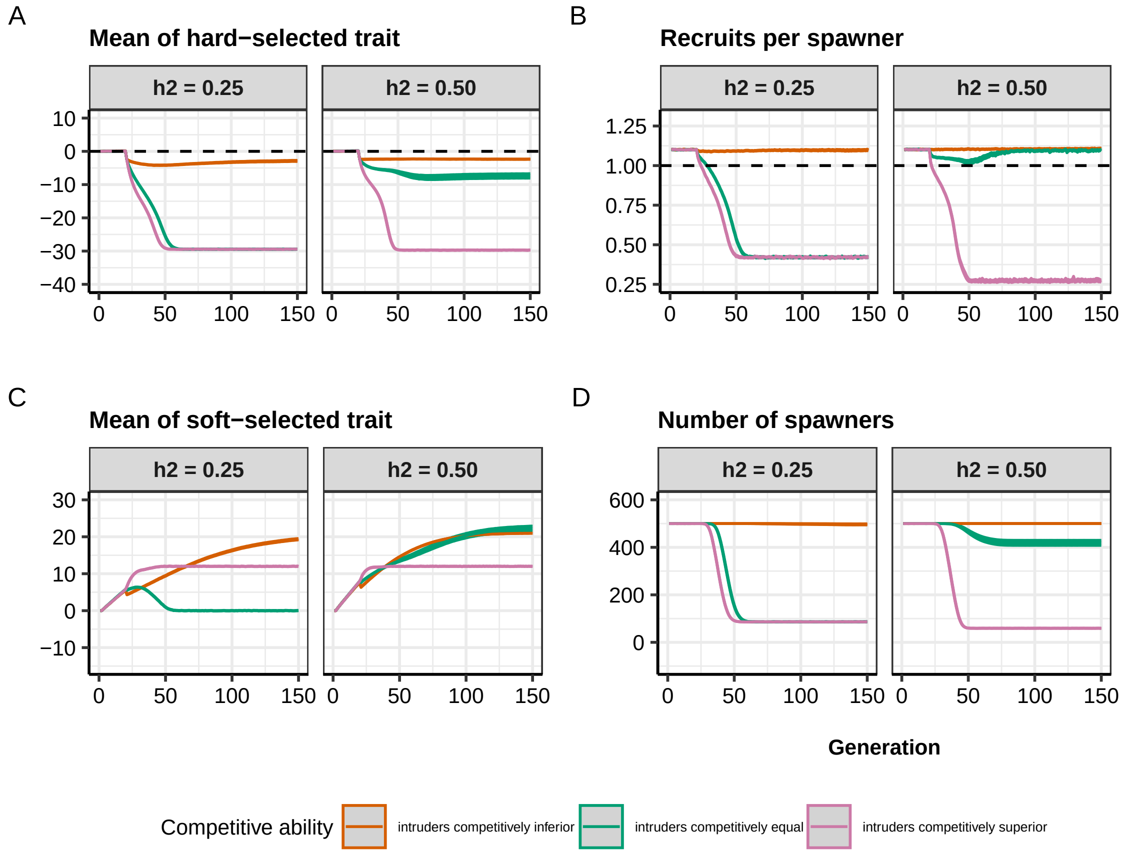
A
Mean of hard−selected trait
h2 = 0.25
h2 = 0.50
B
Recruits per spawner
h2 = 0.25
h2 = 0.50
C
Mean of soft−selected trait
h2 = 0.25
h2 = 0.50
D
Number of spawners
h2 = 0.25
h2 = 0.50
Generation
Competitive ability
intruders competitively inferior
intruders competitively equal
intruders competitively superior
