# Supplementary material for: Competitive interactions affect introgression and population viability amidst maladaptive hybridization
Source: Evol Appl. 2024 Jul 1;17(7):e13746. doi: 10.1111/eva.13746 (PMC11217556; doi:10.1111/eva.13746)
Supplement: Supplementary file 1 — Figures S1–S7. [file EVA-17-e13746-s001.zip › eva13746-sup-0007-Figure_S7.pdf]

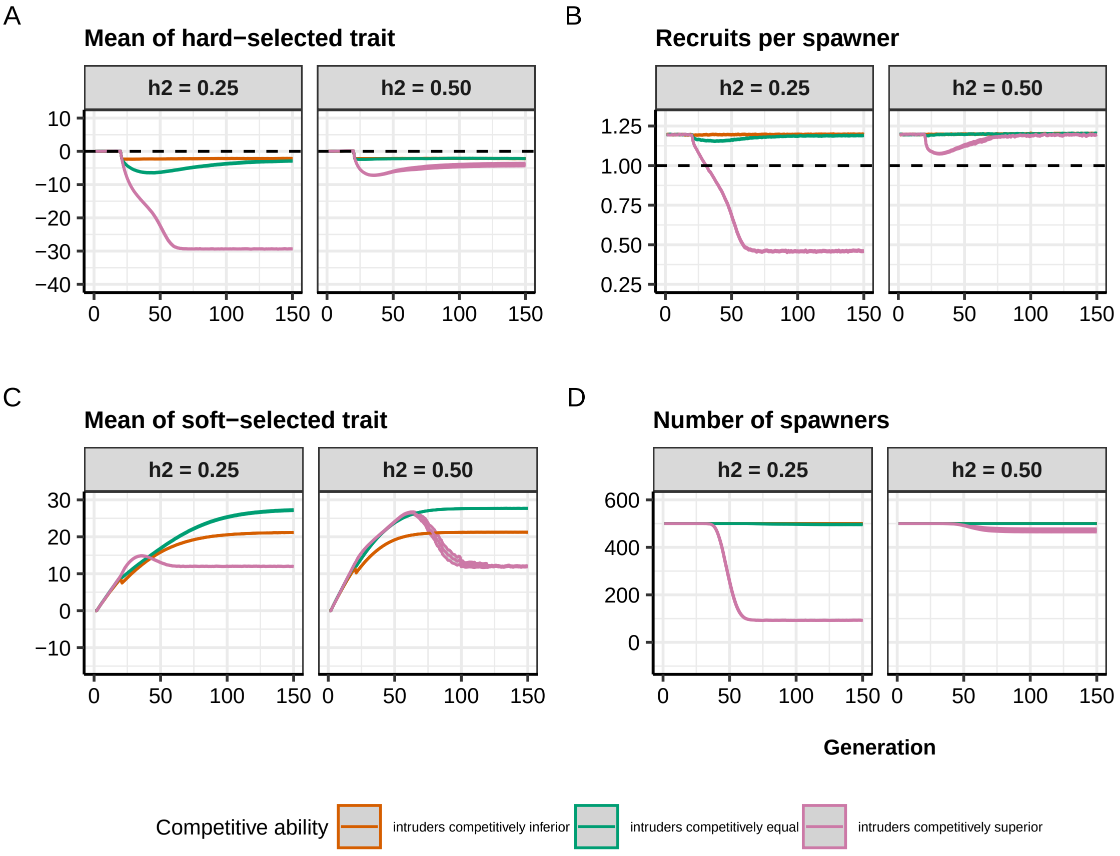
A
Mean of hard−selected trait
h2 = 0.25
h2 = 0.50
10
0
−10
−20
−30
−40
0
50
100
150
B
Recruits per spawner
1.25
1.00
0.75
0.50
0.25
C
Mean of soft−selected trait
30
20
10
0
−10
D
Number of spawners
600
400
200
0
Generation
Competitive ability
intruders competitively inferior
intruders competitively equal
intruders competitively superior
