# Supplementary material for: Competitive interactions affect introgression and population viability amidst maladaptive hybridization
Source: Evol Appl. 2024 Jul 1;17(7):e13746. doi: 10.1111/eva.13746 (PMC11217556; doi:10.1111/eva.13746)
Supplement: Supplementary file 1 — Figures S1–S7. [file EVA-17-e13746-s001.zip › eva13746-sup-0008-supinfo.docx]

Fig.S1: The evolutionary dynamics of $Z_{HARD}$ in the intrusion simulations set 2. K=500 in all scenarios. Low intrusion = 250 intruders introduced in generation 20; moderate intrusion = 500 intruders introduced; high intrusion = 750 intruders introduced.

Fig.S2: Changes in $RPS$ in the acute intrusion simulations set 2. $K$=500 in all scenarios. Low intrusion = 250 intruders introduced in generation 20; moderate intrusion = 500 intruders introduced; high intrusion = 750 intruders introduced. Low reproductive excess: $W_{MAX}=0.53$; moderate reproductive excess: $W_{MAX}=0.58$; high reproductive excess: $W_{MAX}=0.63$. Each panel shows the trajectory of $RPS$ over time, with the average taken each generation over only those replicate populations that persisted ($N_{S}>0$). Initial $h^{2}$= 0.25.

Fig.S3: Probability of extinction in the acute intrusion simulations set 2. $K$=500 in all scenarios. Low intrusion = 250 intruders introduced in generation 20; moderate intrusion = 500 intruders introduced; high intrusion = 750 intruders introduced. Low reproductive excess: $W_{MAX}=0.53$; moderate reproductive excess: $W_{MAX}=0.58$; high reproductive excess: $W_{MAX}=0.63$. Initial $h^{2}$= 0.25.

Fig.S4: Effects of level of maladaptation of intruders on the results of acute intrusion simulations. (A) Evolutionary trajectory of $Z_{HARD}$. (B) Number of spawners through time. Mean and 95% confidence intervals across 1000 replicates shown. In all cases, a moderate level of acute intrusion (500 intruders introduced at generation 20) and a moderate level of reproductive excess ($W_{MAX}=0.58$) was assumed, with initial $h^{2}$= 0.25.

Fig.S5: Results of chronic intrusion simulations set 3 for the low reproductive excess scenario ($W_{MAX}=0.53$). Each panel shows the results (mean and 95% confidence intervals across 1000 replicate simulations) comparing cases where the initial heritability of both $Z_{SOFT}$ and $Z_{HARD}$ was $h^{2}=$0.25 (left sub-panels) or $h^{2}=$0.50 (right sub-panels). The per-generation intrusion rate was fixed at 10% of $K$, i.e., 50 foreign/domesticated fish intruded each generation.

Fig.S6: Results of chronic intrusion simulations set 3 for the moderate reproductive excess scenario ($W_{MAX}=0.58$). Each panel shows the results (mean and 95% confidence intervals across 1000 replicate simulations) comparing cases where the heritability (*h^2^*) of both $Z_{SOFT}$ and $Z_{HARD}$ $=$0.25 (left sub-panels) or$=$0.50 (right sub-panels). The per-generation intrusion rate was fixed at 10% of $K$, i.e., 50 foreign/domesticated fish intruded each generation.

Fig.S7: Results of chronic intrusion simulations set 3 for the high reproductive excess scenario ($W_{MAX}=0.63$). Each panel shows the results (mean and 95% confidence intervals across 1000 replicate simulations) comparing cases where the initial heritability (*h^2^*) of both $Z_{SOFT}$ and $Z_{HARD}$ was $=$0.25 (left sub-panels) or $=$0.50 (right sub-panels). The per-generation intrusion rate was fixed at 10% of $K$, i.e., 50 foreign/domesticated fish intruded each generation.
