# Supplementary figures and images for: Competitive interactions affect introgression and population viability amidst maladaptive hybridization
Source: Evol Appl. 2024 Jul 1;17(7):e13746. doi: 10.1111/eva.13746 (PMC11217556; doi:10.1111/eva.13746)

# Recruits per spawner

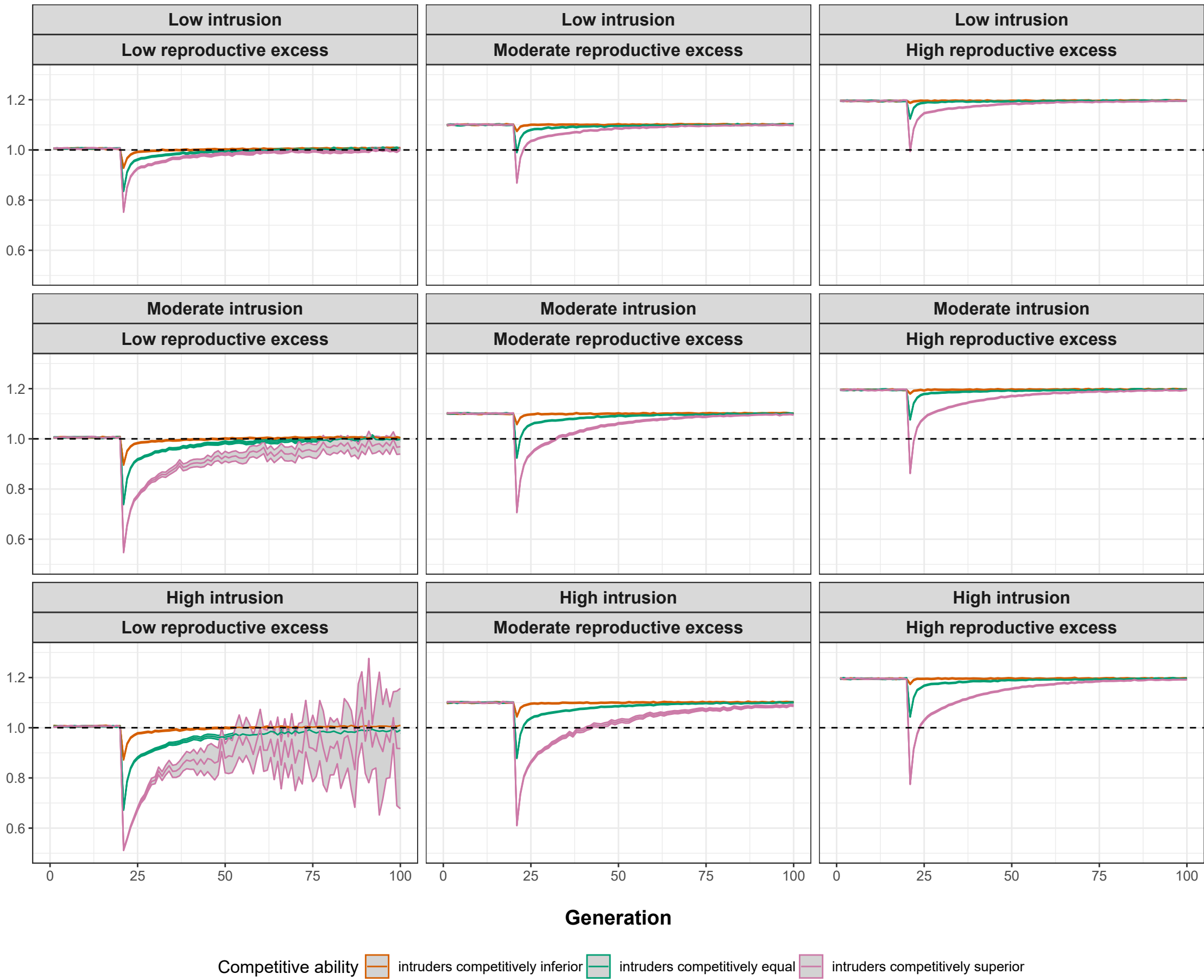

Supplement: Supplementary file 1 — Figures S1–S7. [file EVA-17-e13746-s001.zip › eva13746-sup-0002-Figure_S2.pdf]

**Fraction of replicates that went extinct**

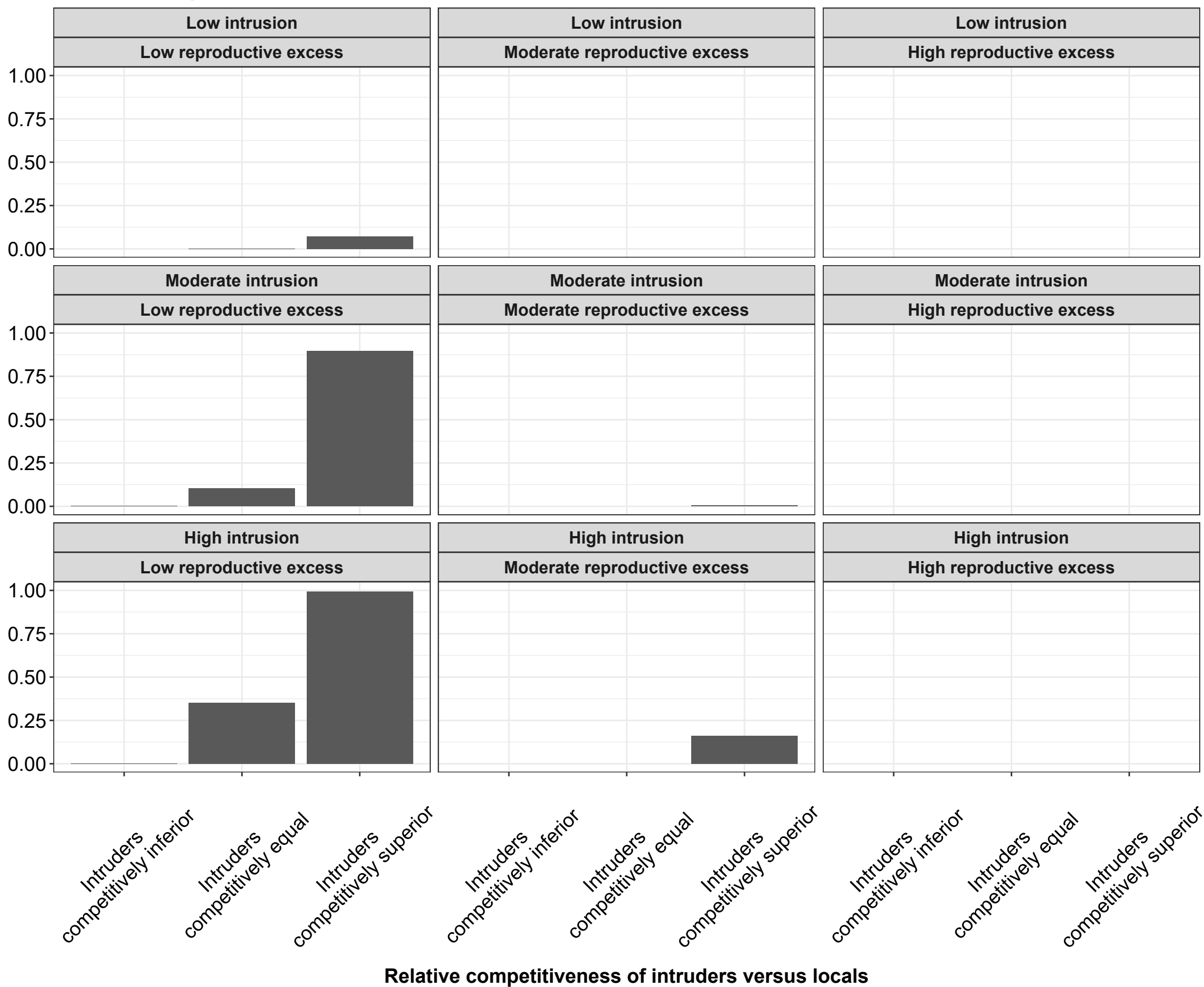

Supplement: Supplementary file 1 — Figures S1–S7. [file EVA-17-e13746-s001.zip › eva13746-sup-0003-Figure_S3.pdf]
